# Adoption challenges to artificial intelligence literacy in public healthcare: an evidence based study in Saudi Arabia

**DOI:** 10.3389/fpubh.2025.1558772

**Published:** 2025-04-30

**Authors:** Rakesh Kumar, Ajay Singh, Ahmed Subahi Ahmed Kassar, Mohammed Ismail Humaida, Sudhanshu Joshi, Manu Sharma

**Affiliations:** ^1^Department of Health Management, College of Public Health and Health Informatics, University of Ha’il, Ha’il, Saudi Arabia; ^2^Department of Management and Information Systems, College of Business Administration, University of Ha’il, Ha’il, Saudi Arabia; ^3^Department of Public Health, College of Public Health and Health Informatics, University of Ha’il, Ha’il, Saudi Arabia; ^4^School of Management, Doon University, Dehradun, Uttarakhand, India; ^5^Department of Management Studies, Graphic Era Deemed to be University, Dehradun, Uttarakhand, India

**Keywords:** artificial intelligence, AI literacy, adoption challenges, public healthcare, Sustainable Development Goals, Saudi Arabia

## Abstract

In recent years, Artificial Intelligence (AI) is transforming healthcare systems globally and improved the efficiency of its delivery. Countries like Saudi Arabia are facing unique adoption challenges in their public healthcare, these challenges are specific to AI literacy, understanding and effective usage of AI technologies. In addition, cultural, regulatory and operational barriers increase the complication of integrating AI literacy into public healthcare operations. In spite of its critical contribution in enabling sustainable healthcare development, limited studies have addressed these adoption challenges. Our study explores the AI literacy adoption barriers in context to Saudi Arabian public healthcare sector, focusing on its relevance for advancing healthcare operations and achieving Sustainable Development Goals (SDGs). The research aims to identifying and addressing the adoption challenges of Artificial Intelligence literacy within the public healthcare in Saudi Arabia. The research aims to enhance the understanding of AI literacy, its necessity for enhancing healthcare operations, and the specific hurdles that impede its successful AI adoption in Saudi Arabia’s public healthcare ecosystem. The research employs a qualitative analysis using the T-O-E framework to explore the adoption challenges of AI literacy. Additionally, the Best-Worse Method (BWM) is applied to evaluate the adoption challenges to AI literacy adoption across various operational levels within Saudi Arabia’s public healthcare supply chain. The study uncovers substantial adoption challenges at operational, tactical, and strategic level, including institutional readiness, data privacy, and compliance with regulatory frameworks. These challenges complicate the adoption of AI literacy in the Saudi public healthcare supply chains. The research offers critical insights into the various issues affecting the promotion of AI literacy in Saudi Arabia’s public healthcare sector. This evidence-based study provides essential commendations for healthcare professionals and policymakers to effectively address the identified challenges, nurturing an environment beneficial to the integration of AI literacy and advancing the goals of sustainable healthcare development.

## 1 Introduction

**Figure 1:**
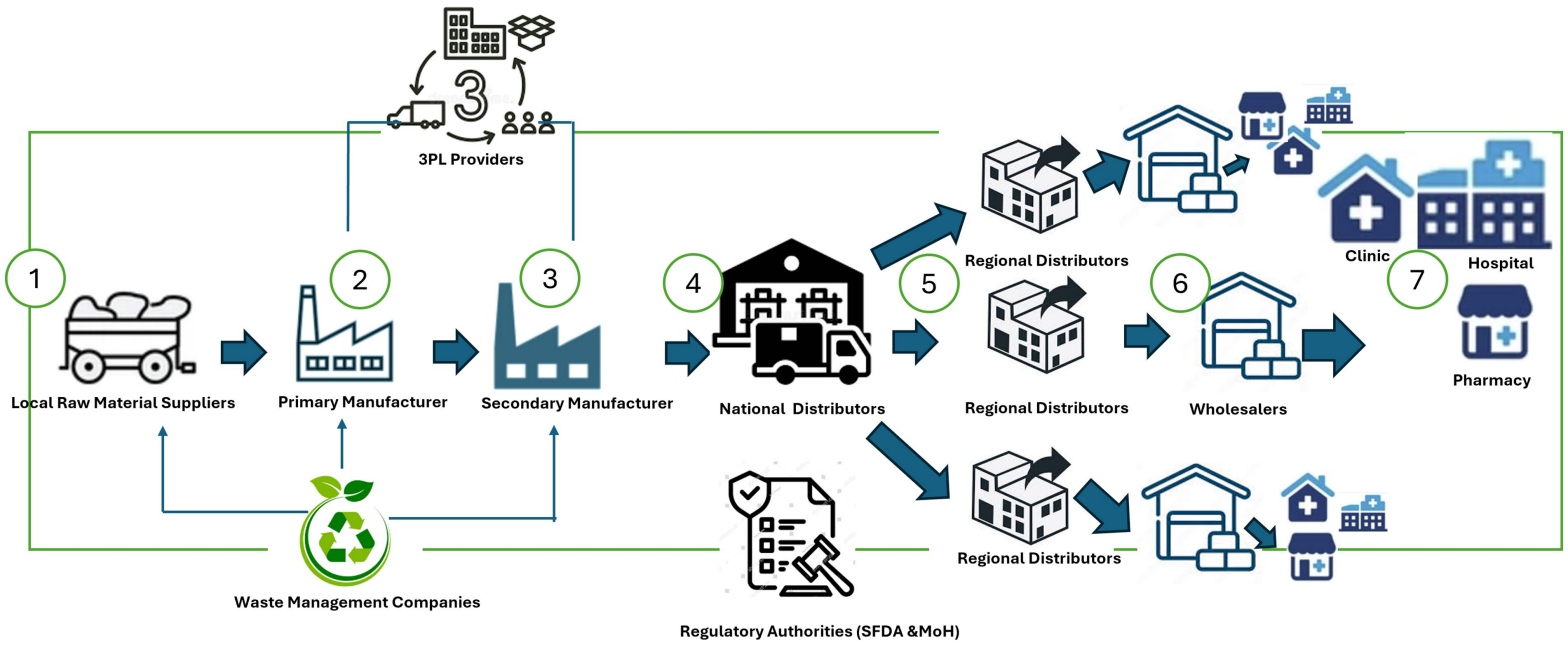
Public healthcare system (PHS) (Source: Authors).

Artificial Intelligence (AI) is reshaping public healthcare systems, enhancing the prediction, treatment, and management of health conditions (1). In line with Vision 2030, Saudi Arabia is committed to advancing technological integration, particularly AI, within its public healthcare, offering substantial promise while facing formidable challenges (2). As depicted in Figure 1, A typical public healthcare system (PHS) consists of key Supply Chain players, including Local Raw Material Suppliers, Primary and Secondary Manufacturers (medical suppliers, pharmaceuticals, essential healthcare equipment makers), Logistics providers, National and Regional Distributors, public health service providers (Government hospitals and clinics) and essentially the regulatory bodies (including SFDA and MoH) (3).

To unleash the capacity of AI in enhancing healthcare delivery, it is critical to identify and develop strategies that promote AI literacy among critical stakeholders, addressing the challenges hindering its effective implementation (4, 5). AI has tremendous potential to analyze electronic health data, uncover patterns, and offer valuable insights for decision-making (6, 7). Traditionally, the healthcare industry generates vast amounts of data through clinical health records, image data, and patient monitoring (8, 9). Over the decades, AI literacy in healthcare supply chains has evolved significantly while harnessing the data to improve diagnostics, treatments, and patient outcomes (10). In the 1950s, basic automation and rule-based logistics and inventory management systems of healthcare inventory were introduced (11). By the 1990s, the rise of computing technologies and the internet facilitated the digitalization of supply chain processes. Also, it introduced the need for AI literacy in areas like telemedicine, logistics and decision support systems (11). In the 2000s, the adoption of Electronic Health Records (EHR) enabled healthcare supply chains to analyze large datasets for demand forecasting and resource optimization (11). By the 2010s, AI literacy expanded to include knowledge of data decision making, which became essential for efficiency and responsiveness in the Supply Chains (10, 11). Today, AI literacy in public healthcare encompasses advanced digital technologies for managing complex logistics, predictive analytics for inventory and demand planning, and the ethical use of AI to ensure transparency, mitigate bias, and maintain data privacy in the increasingly interconnected public healthcare ecosystem (12–14). Table 1 explained the evolution of AI Literacy.

**Table 1:** Evolution of AI literacy.

Stage	Description	Key applications	Use cases	Firms
Basic automation (1950s–1980s)	Introduction of basic automation and rule-based systems for logistics and inventory management in Public healthcare (139, 140).	Rule-based inventory control (139) Logistics automation (141).	Automated inventory replenishment in hospitals (139). Basic supply management systems for pharmacies (141, 142).	IBM (early automation systems) GE Healthcare.
Digitalization of processes (1990s)	Computing technologies and the internet enabled digital procurement and distribution systems enabled PHS (143). Introduction of telemedicine logistics and decision support systems (144).	Telemedicine logistics Automated procurement and distribution (39).	Remote delivery of medical supplies through telehealth networks (39, 144). Automated procurement platforms for hospital chains (145).	Oracle (procurement systems) Siemens Healthineers
Electronic health records & ML adoption (2000s)	Widespread adoption of EHR enabled PHS to store and analyze large datasets (146, 147). Machine learning applications emerged for demand forecasting and resource optimization (148).	EHR data analytics (146) Demand forecasting and resource management (148).	Predicting shortages of essential medicines through EHR analytics (146, 147). Optimizing staff and resource allocation in hospitals based on patient data (149).	Cerner (EHR management) Epic Systems (resource management).
Big data and predictive analytics (2010s)	AI literacy expands to big data and predictive analytics (150). These tools improved efficiency and responsiveness in healthcare supply chains (151).	Predictive analytics for healthcare inventory (152) Real-time decision support systems (152).	Using predictive models to prevent stockouts (152). Real-time decisions for distributing emergency medical supplies (153).	Palantir (predictive analytics) SAP (supply chain systems).
Advanced AI integration (2020s–present)	Advanced AI technologies are now integrated into healthcare logistics (154). AI literacy now includes knowledge of ethical AI use, data privacy, and bias mitigation (155).	NLP (Natural Language Processing)-powered logistics (156) Predictive analytics for demand planning (152) Computer vision for public Health supplies monitoring (157).	Predicting demand spikes for vaccines using AI-powered analytics (152). Ensuring ethical AI practices by monitoring data usage and minimizing algorithmic bias (155). Computer vision systems for tracking medical supplies (157).	Amazon Web Services (NLP tools) Philips (AI-based analytics)

AI literacy has recently advanced beyond basic automation comprehension, integrating big data analytics for predictive insights (15, 16). This progression has profoundly impacted PHS through data-driven decisions, predictive resource allocation, and improved patient outcomes (16). Identically, AI literacy significantly enhances operational and Supply Chain activities across various sectors while better adoption and implementation and strengthening the capabilities of users. In traffic management, the Increasing understanding of AI optimizes routing and reduces congestion, improving logistics efficiency (4, 17). For smart cities, AI-driven systems manage energy, water, and waste resources, enhancing sustainability (18). In e-commerce, AI literacy enables predictive analytics for demand forecasting, inventory management, personalized customer experiences, driving efficiency (19). In the FMCG (Fast-moving consumer goods) sector, AI helps optimize stock levels, reduce waste, and streamline procurement processes (20). In education, AI literacy can support automated distribution, optimize campus logistics, and improve operational efficiency using data-driven decision-making (21). AI literacy can significantly influence the pharmaceutical sector by enhancing the effectiveness in drug distribution, cold chain management, and regulatory compliance (22). Public administration benefits from AI in resource allocation and disaster response logistics, improving public services delivery (23). In public services, AI literacy supports supply chain transparency, demand forecasting, and efficient distribution of critical resources, ensuring effective governance and service delivery (24). Table 2 demonstrates role of AI literacy in improving the operational efficiency in various industries.

**Table 2:** AI literacy and its influence on operational efficiency in various industries.

Sector	Description	Key applications	Use cases	Firms
Traffic management	Increasing understanding of AI optimizes routing and reduces congestion, improving logistics efficiency (36, 158).	AI-driven routing systems Congestion management tools.	Real-time traffic flow optimization using AI algorithms (159). Reduction of travel time through smart routing.	Waycare (traffic management solutions)
Smart cities	AI-driven supply chains manage resources like energy, water, and waste, enhancing sustainability (159).	Resource management systems Sustainability analytics.	Smart waste management systems that optimize collection routes (160, 161). Energy consumption optimization in public utilities.	Siemens (smart city solutions)
E-commerce	AI literacy enables personalized customer experiences (162, 163).	Predictive inventory systems Personalized recommendation engines.	Demand forecasting models to prevent stockouts and overstock (164). Customized shopping experiences based on user data analytics (165).	Amazon (e-commerce logistics)
FMCG sector	AI helps optimize stock levels, reduce waste, and streamline procurement processes (166).	Stock optimization tools Waste reduction analytics.	Automated inventory management systems that adjust stock levels in real time (167). Procurement systems that analyze purchase patterns to minimize waste (168).	Procter & Gamble (FMCG supply chain)
Education	AI literacy supports automated distribution, optimizing campus logistics, and improving operational efficiency through data-driven decision-making (150).	Campus logistics management systems Automated resource distribution tools.	Optimizing the distribution of learning materials and resources on campus (169). Data-driven scheduling systems for facility management.	Blackboard (educational logistics solutions)
Pharmaceutical sector	AI literacy enhances drug distribution, cold chain management, and regulatory compliance (59).	AI-powered drug tracking systems Cold chain monitoring tools.	Predictive analytics for optimizing drug distribution routes (170). Real-time monitoring of temperature-controlled shipments to ensure drug integrity (171).	Pfizer (drug distribution systems)
Public administration	AI benefits resource allocation and disaster response logistics, improving public service delivery (172).	Resource allocation analytics Disaster response management systems.	Efficient allocation of emergency services during disasters (172). Real-time data analysis for optimizing public resource distribution.	Civis Analytics (public service solutions)
Public services	AI literacy supports supply chain transparency, demand forecasting, and efficient distribution of critical resources, ensuring effective governance and service delivery (40).	Supply chain transparency tools Demand forecasting systems.	Ensuring timely distribution of critical supplies during emergencies (173). Analytics to predict resource needs in various public service sectors.	IBM (public service analytics)

Thus, across all sectors, even with slow adoption, organizations can enhance operations, optimize costs, and enhance decision making. The AI advancements are particularly relevant in Saudi Arabia to implement AI-driven solutions, aligning with Sustainable Development Goals (SDGs) to enhance healthcare sustainability, efficiency, and equity (25, 26). However, challenges remain in ensuring widespread AI literacy among stakeholders to leverage these technologies for sustainable public health improvements fully. Thus, the research aims to address research questions and objectives are as follows:

**RQ1:** What are the major adoption challenges to AI literacy in PHS, in Saudi Arabia?

**RO1:** To identify the key adoption challenges while adopting AI in PHS, in Saudi Arabia.

**RQ2:** What are the extremely critical AI literacy adoption challenges in PHS, in Saudi Arabia?

**RO2:** To identify extremely AI adoption challenges and discuss the roadmap to reduce AI adoption barriers in PHS, in Saudi Arabia.

**RQ3:** What is the strategic roadmap to AI adoption in PHS.

**RO3:** To suggest the framework for exploring AI adoption challenges, emphasizing the prioritization of critical barriers and strategic roadmap development.

The organization of the research paper as: Section 2 provides comprehensive review on AI literacy within Public Healthcare System, examined through the lens of SDGs, and presents the foundational theories underpinning the study. Section 3 explained methodology employed in the current research. Section 4 introduces a comprehensive framework for AI implementation. Section 5 details the main outcomes of the study. Section 6 proposes a strategic roadmap to mitigate the challenges hindering AI adoption in PHS. Finally, Section 7 explains limitations and highlights areas for further studies.

## 2 Literature review

The Systematic Literature Review (SLR) has been carried out on relevant literature from Scopus database. SLR process conducted using multiple keywords, including “AI Literacy,”; “Public Healthcare,”; “Public Healthcare System,”; “Saudi Arabia,” and “Sustainable Development Goals.” The SLR used a timeline between 2019 and 2024. The search yielded 518 articles. Table 3 depicts the search protocol used for this study.

**Table 3:** Search protocol deployed for the study.

Key elements	Description
Keywords	“AI Literacy,”; “Public Healthcare,”; “Public Healthcare System”; “Saudi Arabia,” and “Sustainable Development Goals.”
Timeline	2019–2024
Field analyzed	Research title, Full Abstract, Keywords, Document types
Inclusion criteria	Scopus listed documents
Exclusion criteria	Non-English Articles

In the first screening results into the removal of duplicate articles, 352 publications that meet with the research objectives. Further, in next level of screening, working papers, conference papers, and conference proceedings were also omitted and 183 articles where retrieved. After further investigation in the third screening, it was determined that 73 papers were pertinent to the study questions. In the fourth screening A cross-referencing method was used to finalize the paper selection process, and 37 papers were ultimately chosen. The SLR procedure used by the authors for this investigation (Table 4).

**Table 4:** Systematic literature review (SLR) process.

Search term	First screening results	Second screening results	Third screening results	Fourth screening results
“AI Literacy” AND “Healthcare”	126	68	43	26
“Health Supply Chain Management” AND “Artificial Intelligence”	56	36	18	2
“Saudi Arbia” AND “Sustainable Development Goals” AND “Healthcare”	170	78	12	9
Total articles				37

### 2.1 AI in public healthcare in Saudi Arabia

In Saudi Arabia, development of AI in public services is primarily driven by the Vision 2030 initiative (26). The key aim of Vision 2030 is to transform and develop the country toward a digitally empowered, diversified, self-reliant and sustainable economy (27). The application of AI in the government has helped in enhancing government services and key decision-making processes and efficiency in the allocation of the available resources (28). Recent strategic initiatives in Saudi Arabia, viz., Vision 2030, were inducted to achieve United Nation’s SDGs and to drive the AI adoption process in various public sectors (29). The National Strategy uses AI for economic growth, healthcare improvements, environmental sustainability, and better citizen services, aligning with Vision 2030 goals (30). These strategic goals aim to project Saudi Arabia as a global AI leader by 2030 while emphasizing data-driven approaches for governance and public services for the masses (31, 32). AI is increasingly adopted across Saudi Arabia’s PHS to enhance patients’ treatment, diagnostic methods, and hospital administration (30). Table 5 depicts various potential areas of AI applications in PHS.

**Table 5:** AI applications in public healthcare.

Category	Application	Description	Use-cases from Saudi Arabia	Benefits
Diagnostics	Enhanced Imaging Analysis	Artificial intelligence algorithms utilize advanced computational techniques to examine medical images (174).	Identifying cancerous lesions in mammograms or retinal diseases in eye scans (175)	Optimizing imaging equipment supply and maintenance scheduling (176).
			King Faisal Specialist Hospital & Research Centre uses AI to enhance radiology and pathology imaging (177).	
	Pathology	AI assists pathologists in analyzing tissue samples, improving speed and precision in diagnosing cancer (178, 179)	AI systems are used in hospitals like King Saud Medical City to improve cancer diagnosis accuracy (180)	Automating sample logistics between collection centers and labs (181).
Personalized medicine	Genomic Analysis	AI analyzes genetic data to identify mutations and predict an individual’s response to treatments (182).	Saudi Human Genome Program utilizes AI to analyze genetic data for personalized treatment plans (183)	Managing genomic sample storage and delivery in research workflows (184).
	Predictive Analytics	AI predicts patient outcomes based on historical data, allowing for tailored treatment plans (185, 186).	AI-driven predictive analytics in hospitals like King Abdulaziz Medical City to tailor treatment (8)	Demand forecasting for personalized medication supply (187).
Patient care	Remote Monitoring	AI-powered wearables and sensors monitor patients’ vital signs, alerting providers to potential issues (188)	The Ministry of Health uses AI-powered remote monitoring for patients with chronic conditions (189)	Ensuring timely replenishment of wearable devices and sensors (188).
	Telehealth	AI enhances telehealth by providing virtual consultations and remote diagnostics, increasing accessibility (190).	AI-enabled telehealth platforms (191)	Streamlining telemedicine kit distribution to remote areas during COVID-19 [96].
Administrative efficiency	Medical Record Management	AI automates the extraction and organization of data from EHRs, reducing administrative burdens (192).	Hospitals in Riyadh are adopting AI for efficient EHR management and data organization (193)	Efficient management of medical record archiving and retrieval (194).
	Revenue Cycle Management	AI improves billing processes by identifying coding errors, optimizing claims, and predicting denials (195).	AI is being utilized in Saudi hospitals to streamline billing processes and reduce errors in claims (196)	Optimizing supply chain for claim documents and billing systems (197).
Research and drug development	Drug Discovery	Artificial intelligence speeds up discovering new drugs by analyzing extensive datasets (198).	AI research centers in Saudi Arabia, like KAUST, accelerate drug discovery and development (199)	Coordinating logistics for drug compounds and R&D materials (200).
	Clinical Trials	AI helps design and manage clinical trials efficiently, selecting participants and monitoring outcomes (201).	AI is used in clinical trials for new medications at King Fahd Medical City, improving participant selection (202)	Managing participant supplies and trial site inventory (203).
Predictive healthcare	Early Detection of Diseases	AI models leverage patient data to detect initial indications of diseases, facilitating prompt intervention (204).	AI is deployed in early detection programs for diabetes and heart disease across Saudi Arabia (205)	Coordinating early screening kits and diagnostics supply chains (206).

### 2.2 Relevance of AI literacy for sustainable performance of public healthcare system

AI literacy becomes relevant for sustainable performance of public healthcare, especially with the adoption of AI technologies with the aim to optimize operations and sustain performance (33). AI literacy among stakeholders (including policymakers and supply chain managers) ensures responsible usage of AI (34). The recent advancement emphasized focusing on AI literacy to improve decision-making in supply chains. AI can enhance SC performance by predicting demand, optimizing inventory, and identifying bottlenecks, ultimately reducing costs and waste (35). In healthcare logistics, using deep reinforcement learning models substantially improved economic, environmental and social outcomes while selecting sustainable chain modes more efficiently than traditional methods (36). Additionally, AI literacy significantly mitigates data-driven risks, including algorithmic bias, privacy infringements, and poor data governance. Such issues are critical for AI-driven systems to align with patient-centric and ethical standards (37). In addition to data-driven decision-making, the role of AI literacy in catering to public health crisis activities, including improving resource distribution during emergencies by leveraging underlined technologies (38). Also, using digital AI literacy using predictive algorithms and big data analytics in the healthcare supply chain as its consumption reduces the error rate and enhances productivity (39, 40). Public healthcare systems use multiple literacy programs that aim at improving digital and health AI competencies, including familiarity with predictive algorithms and big data analytics, which are necessary to foster trust and smooth integration of AI in healthcare settings. Through AI adoption, public healthcare systems remain sustainable and adaptive to evolving challenges (41).

The growing need for AI literacy is also linked to the ability to interpret AI-driven insights accurately and act upon them (42). Therefore, embedding AI literacy as a critical skill among healthcare professionals and supply chain managers will be vital in driving sustainable performance in public healthcare systems (43). These developments underline the importance of continuous training and strategic frameworks to improve AI literacy, ensuring the long-term success of public healthcare systems (44). The significance of taking interests of all parties involved in organization’s activities is emphasized by stakeholder theory (45). In the context of public healthcare systems, AI literacy becomes critical for ensuring sustainable and effective collaboration among stakeholders such as health service providers, policymakers, suppliers, patients, and regulators (10).

(a) **Healthcare providers as stakeholders:** AI literacy among healthcare professionals ensures they can leverage AI-driven tools to enhance decision-making, streamline inventory management, and predict resource needs effectively. With better understanding, providers can work collaboratively to use AI for predictive diagnostics, patient data management, and optimizing logistics during emergencies, fostering sustainable healthcare delivery (46).
(b) **Policymakers and regulators:** Policymakers participation has a crucial role in creating frameworks that ensure the responsible use of AI. An AI-literate policymaker can promote transparency, data privacy, and address ethical concerns around AI-driven healthcare systems through a sustainable policy framework. AI literacy enables stakeholder alignment by bridging communication gaps between technical teams, regulators, and healthcare practitioners, ensuring AI solutions are both compliant and practical (47).
(c) **Patients and the public:** Patients, as indirect stakeholders, benefit from AI-driven supply chains through improved service delivery, faster diagnostics, and better resource availability. AI literacy among the public encourages trust in AI tools and ensures acceptance of innovations such as AI-powered telemedicine platforms or automated drug delivery systems (48).
(d) **Suppliers and partners:** AI-literate suppliers can optimize their processes by utilizing predictive analytics for inventory planning, reducing waste, and ensuring timely deliveries (49). AI literacy enhances cold chain management in the pharmaceutical sector, ensuring that temperature-sensitive products are delivered effectively and sustainably.
(e) **Governance and collaboration among stakeholders:** AI-driven governance mechanisms that align the interests of multiple stakeholders are critical for the sustainability of healthcare supply chains (50). AI literacy fosters collaborative efforts between public and private stakeholders to ensure efficient logistics management during public health emergencies (51). For example, understanding predictive algorithms and big data analytics allows stakeholders to collaborate effectively on disaster response logistics and resource allocation (52).
(f) **Social and environmental impact:** AI literacy helps healthcare stakeholders align their operations with broader sustainability goals by reducing carbon footprints through optimized transportation and supply chain routes (53). It enables organizations to adopt circular economic practices, ensuring waste management through effective resource utilization.

AI literacy within and among healthcare stakeholders strengthens sustainable performance by ensuring all parties can collaborate effectively. It mitigates algorithmic bias, poor data governance, and compliance risks. Furthermore, it helps align stakeholders’ interests toward better patient outcomes, resource optimization, and environmental sustainability, ensuring public health systems are resilient, adaptive, and equitable (54).

### 2.3 Rise of AI literacy

A surge in AI literacy facilitates the use of AI tools in PHS in Saudi Arabia (55). Beyond operational benefits, AI literacy aligns with the SDGs driving healthcare innovation and contributing to sustainable development in several ways (56). AI literacy empowers healthcare professionals and supply chain managers to utilize advanced AI tools effectively, enhancing healthcare supply chains across multiple dimensions (33). It enables predictive analytics, demand forecasting, and inventory management to prevent stockouts or overstocking, ensuring optimal resource utilization (57). Automated AI-driven systems reduce human errors and provide real-time monitoring, ensuring timely delivery of essential supplies, even to remote or underserved areas (58). Additionally, AI integrated with blockchain enhances transparency and traceability in tracking pharmaceuticals and vaccines, mitigating counterfeit risks and safeguarding patient safety (59). Furthermore, AI literacy promotes sustainable operations by minimizing waste, improving recycling, enabling energy-efficient logistics, driving cost-effectiveness and reducing the sector’s carbon footprint in alignment with broader sustainability goals (60). AI literacy aligns with multiple SDGs by enabling stakeholders to leverage AI for sustainable healthcare development (61). It supports SDG 3 (Good Health and Wellbeing) by optimizing the availability of essential medical supplies, enhancing healthcare outcomes, and ensuring effective responses to public health crises across Saudi Arabia (55). In line with SDG 9, AI literacy fosters innovation and resilient healthcare infrastructure, advancing the digital transformation goals of Vision 2030 (62). It also promotes SDG 12 by enhancing resource optimization, reducing waste, and encouraging sustainable use of pharmaceuticals and medical equipment (63). By creating high-skilled jobs in healthcare technology and logistics, AI literacy contributes to SDG 8, driving economic diversification and operational efficiency (64). Additionally, AI literacy advances SDG 17 by facilitating collaborations among technology providers, healthcare organizations, and government agencies, ensuring scalable AI solutions for local and global healthcare systems (65). There are some major challenges that need to be raised concerning the general concept and practice of AI literacy (35). Professionals must understand data privacy and security measures to safeguard sensitive health information, aligning AI use with regulatory frameworks and SDG privacy standards (66). Additionally, adapting AI systems to Saudi Arabia’s cultural context and regulatory environment requires literacy in both AI technology and local healthcare laws to ensure smooth integration into clinical workflows (67). AI literacy should also encompass knowledge of the environmental impact of AI systems, such as energy consumption, to align adoption with SDG 13, promoting a balance between innovation and sustainability (68). Development of AI literacy among healthcare professionals, supply chain managers, and policymakers enables Saudi Arabia to unlock the full potential of AI-powered public health systems, improving healthcare services while advancing the country’s contribution to the SDGs (12). Saudi Arabia’s Vision 2030 offers a strategic framework for the sustainable integration of AI solutions, ensuring healthcare transformation aligns with global development objectives (55). Emphasizing AI literacy ensures that these technologies drive more than just automation, promoting informed decision-making, sustainable consumption, and economic growth while safeguarding societal wellbeing and environmental sustainability (69). The rise of AI in Public Healthcare Systems in Saudi Arabia presents significant potential for advancing the country’s healthcare sector while contributing to Sustainable Development Goals (SDGs) (70). AI enhances the efficiency of operations through predictive analytics, demand forecasting, and inventory management. By predicting the demand for medical supplies and pharmaceuticals, AI helps avoid shortages or overstocking, optimizing resource utilization (48, 71). AI-powered automation reduces manual errors and enables real-time monitoring of supply chain processes. This ensures that critical supplies reach hospitals and healthcare centers promptly, particularly in remote or under-served areas (72). AI combined with blockchain technology to ensures transparency in the supply chain, particularly for pharmaceuticals and vaccines. This is critical in preventing counterfeit drugs and ensuring patient safety (72). AI helps minimize waste by analyzing data on consumption patterns, improving recycling, and implementing energy-efficient logistics. It contributes to lowering carbon footprints and healthcare costs (73).

AI literacy, use Artificial Intelligence for transforming healthcare supply chains in ways that align with sustainable development (74). Empowering healthcare professionals, policymakers, and supply chain managers with AI knowledge ensures that advanced tools are adopted strategically, enhancing healthcare systems, improving sustainability, and advancing the United Nations SDGs (75). AI literacy enables healthcare personnel to use AI enabled algorithms in demand forecasting for medical supplies, vaccines, and pharmaceuticals (76). Informed decisions based on AI insights ensure consistent availability of essential drugs and equipment, reducing stockouts and delays (48). With AI tools integrated into the supply chain, professionals can act faster and more effectively in public health crises, ensuring a high standard of care across the country (77). Understanding how AI can improve supply chain operations empowers healthcare leaders to adopt innovations like real-time tracking, predictive maintenance, and automated inventory systems (78). AI literacy enables organizations to optimize resource planning, leading to more resilient healthcare infrastructure (79). In alignment with Vision 2030, AI-literate professionals can spearhead digital transformation, positioning Saudi Arabia as a hub for healthcare infrastructure development (80). AI-literate supply chain managers can leverage machine learning models to minimize waste and promote responsible use of medical resources (81). By accurately forecasting demand and managing inventory, they avoid overstocking and the expiration of pharmaceuticals, reducing environmental impact. Increased awareness of AI tools also allows professionals to track and recycle equipment, fostering a circular economy in healthcare (82). Developing AI literacy creates pathways for new job roles in healthcare logistics, data analytics, and AI system management (83). As AI automates routine tasks, workers can transition to high-value roles, driving operational efficiency and economic growth (84). The expansion of AI-powered healthcare systems also offers opportunities for collaboration between industries, contributing to the diversification with Vision 2030 (85). AI literacy strengthens multi-stakeholder collaboration by enhancing the ability of healthcare providers, policymakers, and private-sector partners to deploy AI solutions. Informed stakeholders are more capable of fostering public-private partnerships and aligning AI-driven supply chains with national and global development priorities (86). Cross-border knowledge-sharing initiatives are also facilitated by AI literacy, promoting global healthcare advancements (87). By promoting AI literacy across the healthcare supply chain, Saudi Arabia enhances its capacity to achieve the SDGs through sustainable innovation. Trained professionals can effectively harness AI to address healthcare challenges, optimize supply chains, and foster collaboration among stakeholders (88). Vision 2030 provides the strategic direction for building a digitally literate workforce, integrating AI seamlessly into healthcare infrastructure, and promoting long-term sustainability (89). Ultimately, AI literacy ensures that technological advancements in healthcare supply chain management are implemented thoughtfully, fostering economic growth, improving wellbeing, and promoting responsible consumption and production (33).

### 2.4 AI literacy challenges in Saudi Arabia in comparison to other nations

AI literacy adoption in public healthcare varies across nations due to differences in technological infrastructure, regulatory policies, and workforce preparedness. Saudi Arabia, under its Vision 2030 initiative, is making significant strides in AI integration, particularly in healthcare. However, challenges such as a shortage of AI-trained professionals, resistance to change, and concerns about ethical AI implementation persist. Table 6 explains a comparative analysis with other countries provides valuable insights into how these challenges manifest globally.

**Table 6:** Comparative analysis with other countries.

Country	Case study	Key insights & lessons for Saudi Arabia
United States	Mayo Clinic—AI-Driven Clinical Decision Support	The Mayo Clinic has incorporated the use of AI-based CDSS as a tool for the diagnosis and treatment processes among the healthcare practitioners. Structured AI literacy training programs focus on interdisciplinary collaboration between medical practitioners and AI specialists (207). Saudi Arabia can benefit from similar training-focused AI literacy initiatives within its public healthcare sector.
United Kingdom	NHS AI Lab—AI Workforce Upskilling	The NHS AI Lab has launched structured AI training programs to upskill healthcare professionals in practical AI applications, ethical considerations, and regulatory compliance. Saudi Arabia could adopt a government-led AI literacy initiative to bridge knowledge gaps in the healthcare workforce (208).
India	Apollo Hospitals—AI-Powered Telemedicine	AI adoption in India is growing, particularly in telemedicine and diagnostics, where Apollo Hospitals use AI-driven platforms for remote consultations (209). Despite infrastructure limitations, grassroots AI training programs help improve healthcare accessibility. Saudi Arabia could develop AI-driven telemedicine literacy programs to enhance AI adoption in rural and underserved areas.
United Arab Emirates	Dubai Health Authority—AI and Robotics in Surgery	The UAE has invested in AI-powered robotic-assisted surgeries and predictive healthcare analytics. The Dubai Health Authority (DHA) has integrated AI literacy programs into hospital workflows, ensuring that medical practitioners understand and effectively utilize AI-based decision-making tools (210). Saudi Arabia could adopt a similar strategy to enhance AI competency among healthcare professionals, particularly in specialized fields like radiology and surgery.
Singapore	Ministry of Health—AI in Smart Hospitals	Ministry of Health has integrated AI literacy into hospital workflows and medical education, ensuring responsible usage. The country’s National AI Strategy emphasizes continuous professional development in AI skills, particularly in smart hospitals like the National University Health System (NUHS), where AI is used for predictive analytics and automated diagnostics (211, 213, 214). Saudi Arabia can adopt a similar policy-driven approach to institutionalize AI literacy training within its healthcare system.
Estonia	National eHealth Infrastructure—AI and Digital Health	Estonia has developed a comprehensive AI-integrated digital healthcare system, leveraging its eHealth infrastructure to facilitate AI-driven decision-making. AI literacy efforts focus on empowering healthcare professionals through digital competency programs and incorporating AI modules into national medical training curricula (210, 215). Saudi Arabia can draw lessons from Estonia’s digital-first AI literacy approach, ensuring that AI training is embedded at both policy and institutional levels.

Table 7 explained various takeaways for Saudi Arabia.

**Table 7:** Key takeaways for Saudi Arabia.

Key takeaway	Use-cases	Implementation in Saudi Arabia
AI-integrated medical training	NHS AI Lab (UK), Mayo Clinic (USA) (207, 208)	Saudi medical schools and hospitals could introduce AI-Literacy sensitization and capacity building.
Government-backed AI literacy programs	NHS AI Lab (UK) (208)	Structured AI training initiatives led by the government could help upskill healthcare professionals and ensure ethical and efficient AI adoption.
AI-driven telemedicine initiatives	Apollo Hospitals (India) (209)	AI-powered telemedicine literacy programs could improve access to AI-based remote health services.
AI-focused policy frameworks	Dubai Health Authority (UAE) (210)	Saudi Arabia could develop AI-centric healthcare policies to support ethical AI deployment, regulatory compliance, and workforce readiness.

### 2.5 Challenges to AI literacy in public healthcare systems

Building AI literacy in public healthcare systems, especially in Saudi Arabia, presents unique challenges and considerations due to the Saudi Arabia’s rapid adoption of AI as part of its Vision 2030 agenda (90). AI is being positioned as a tool to advance quality of healthcare services (91). Below are key challenges, considerations, and specific use cases from Saudi Arabia, supported by literature. Based on the literature, authors have identified various operational challenges (OC) in building AI literacy, which is the critical enabler for sustainable public health healthcare systems in developing countries, including Saudi Arabia.

**OC1-Data accessibility and quality:** AI systems in healthcare depend heavily on large datasets, yet data quality and accessibility remain challenges (92, 93). Public healthcare systems in Saudi Arabia still face issues with data fragmentation, impeding AI’s effectiveness (94). Improving data governance and ensuring data interoperability across various healthcare facilities will be key to unlocking AI’s potential (95). Saudi Arabia’s Ministry of Health (MOH) has introduced a Health Information Exchange (HIE) system aimed at centralizing patient data, improving data access and quality for AI-driven systems in supply chains (96).

**OC2-Data security concern:** As AI is adopted more widely in Saudi healthcare supply chains, there are growing concerns about data safety (97). AI literacy programs should include cybersecurity training that addresses specific threats related to AI systems. The public health systems include stringent guidelines for protecting healthcare data used by AI systems, ensuring that staff across the supply chain are aware of and follow cybersecurity best practices (98).

**OC3-Integration with existing system:** Public Healthcare in Saudi Arabia often rely on legacy systems that are difficult to integrate with modern AI technologies (67). AI literacy programs should focus on how to gradually integrate AI tools with these existing systems without causing disruption (99). Saudi Arabia’s digitization efforts under Vision 2030 include projects like the “Seha” telemedicine platform, which leverages AI to optimize patient data management, demonstrating how AI can be integrated into existing healthcare infrastructure (100).

**OC4-Customization and context utilization:** AI models developed in other regions may not fit Saudi Arabia’s specific healthcare challenges, especially in the context of its supply chains (101). Training programs should customize AI solutions that account for Saudi Arabia’s unique healthcare requirements and regulations (102). AI models are being customized for the Saudi healthcare system, especially in rural areas where supply chain disruptions are common. Customized AI systems predict and manage medication stock levels in these areas (103).

**OC5-Patient-centric consideration:** AI decisions in healthcare supply chains can have direct implications for patient outcomes, creating ethical concerns if AI systems make errors (104). AI literacy programs must emphasize patient-centered approaches, ensuring that healthcare workers understand how AI can improve patient care through efficient supply chain management (105). AI-driven systems in Saudi Arabia are being used to predict drug shortages and adjust supply chains, accordingly, ensuring that patients receive the medications they need without delay (106). The Table 8 consolidates the challenges, considerations, and specific use-cases from Saudi Arabia to provide a structured overview of building AI literacy in public health systems.

**Table 8:** AI literacy challenges (from T-O-E perspective).

S no.	Challenges	Definition	Considerations	Use cases (Saudi Arabia)
Technological perspective				
OC1	Data Accessibility and Quality (92–96)	Challenge involves ensuring that data is accurate, timely, and available for decision-making processes, which requires technological investments and innovations in data handling and storage systems.	Data fragmentation impedes AI’s effectiveness; improving data governance is essential for AI-driven solutions (92, 93)	Government has implemented the Health Information Exchange (HIE) system to centralize data (94–96)
OC2	Data Security Concern (97, 98)	Ensuring data security is crucial in healthcare	AI systems pose cybersecurity risks, including the potential for data breaches (91).	The Health Sector Cybersecurity Framework (HSCF) provides guidelines for protecting AI-related healthcare data (114).
OC3	Integration with Existing Systems (67, 99, 100)	Integrating AI solutions with legacy healthcare systems poses technological challenges that require compatibility and interoperability between different technologies.	Legacy systems in healthcare are difficult to integrate with AI technologies without disruption (67, 99).	The “Seha” telemedicine platform manage patient data (100).
Organizational perspective				
OC4	Customization and Context Utilization (101–103)	Tailoring technological solutions to fit specific healthcare settings and patient demographics calls for adaptable, flexible technology configurations.	AI models must be adapted to meet Saudi Arabia’s unique healthcare and regulatory challenges (101–103)	Customized AI models are being developed to manage medication stock in rural areas with frequent disruptions (103).
OC5	Patient-Centric Consideration (104–106)	Ensuring the organizational approach remains patient-centered, adapting processes to prioritize patient outcomes in all AI-driven implementations.	AI decisions can directly affect patient outcomes, raising ethical concerns when errors occur (104, 105).	AI is used to predict drug shortages, helping adjust supply chains to meet patient needs without delays (106).
OC6	Interdisciplinary collaboration (44, 90)	Regulatory requirements come from external governmental and health bodies, impacting how healthcare organizations implement AI within legal and ethical standards.	Collaboration between stakeholders is essential for effective AI adoption (90).	The National Center for Artificial Intelligence (NCAI) facilitates interdisciplinary collaboration on AI projects (44).
OC7	Cost and Resource Allocation (107–109)	The organization must allocate budgets effectively for AI initiatives while balancing cost constraints and revenue potential to support AI in healthcare.	Substantial investments are needed for AI tools, training, and infrastructure (107).	The MOH partners with private companies to implement AI solutions in logistics, enhancing inventory management (108, 109).
OC8	Cultural Resistance and Change Management (110–112)	Organizational resistance to AI integration due to entrenched cultural practices and the need for structured change management to align staff with new technologies.	There is resistance to AI adoption due to fears of job displacement and mistrust in AI algorithms (78).	King Faisal Specialist Hospital has integrated AI to assist with tasks like scheduling and logistics (79).
OC9	Workforce Training and Knowledge Gaps among Health Staff (114–116, 118)	Organizational challenge of training healthcare staff to bridge knowledge gaps, necessitating dedicated programs and initiatives within the organization.	Healthcare professionals in Saudi Arabia often lack AI skills, limiting the effectiveness of AI integration (75).	The National Health Information Center offers AI training programs for healthcare professionals (76).
Environmental perspective				
OC10	Sustainability and Scalability (90, 122)	Technological systems must be designed for long-term sustainability and scalability, especially in settings where resource availability varies.	Concerns about patient privacy and data (90, 122, 212)	SDAIA ensures ethical AI use with regulations like HSCF (122, 216).
OC11	Ethical and Regulatory Perspectives (119–121)	Compliance with ethical guidelines and regulatory standards requires organizational policies and practices to ensure adherence across all AI applications.	Concerns about patient privacy and data security are prevalent, especially with AI’s role in sensitive data (119, 120).	The Saudi Data and AI Authority (SDAIA) ensures ethical AI use with regulations like HSCF (121).

**OC6-Interdiscplinary collaboration:** AI literacy in Saudi Arabia’s healthcare sector requires not only technical knowledge but also collaboration between stakeholders (90). AI literacy efforts must promote interdisciplinary knowledge exchange between them to ensure sustainable health systems (44). The National Center for Artificial Intelligence (NCAI) fosters collaboration between healthcare institutions and AI companies, ensuring that supply chain innovations are driven by both medical expertise and AI technology (44).

**OC7-Cost and resource allocation:** AI implementation in Saudi Arabia’s public health systems requires substantial investments, especially for training, infrastructure, and AI tools (107). Government funding and public-private partnerships are essential to developing AI literacy and sustaining AI initiatives across the healthcare system (108). The MOH in Saudi Arabia has partnered with private tech companies to implement AI-based solutions in healthcare logistics, ensuring more efficient inventory management, while also training staff to utilize these tools (109).

**OC8-Cultural resistance and change management:** Resistance to AI adoption is common among healthcare professionals in Saudi Arabia, particularly due to concerns over job displacement and mistrust in AI algorithms (110). AI literacy programs need to emphasize AI’s role to assist public healthcare partners (111). In Saudi hospitals, AI is gradually being used to assist with repetitive tasks, such as scheduling and supply chain logistics (112). King Faisal Specialist Hospital and Research Centre has integrated AI-powered systems that automate administrative tasks, leading to cost reductions and improved efficiency (113).

**OC9-Workforce training and knowledge gaps among health staff:** Many healthcare professionals in Saudi Arabia lack the necessary AI skills (114). As AI becomes more integrated into healthcare supply chains, professionals must understand how AI models function and how they can be leveraged for tasks like inventory management and demand forecasting (115). Saudi Arabia has initiated training programs to enhance AI literacy among healthcare professionals as part of broader digital health strategies (116, 117). The Saudi Arabia has launched initiatives such as the National Health Information Center to provide training on AI applications in healthcare, emphasizing AI-based decision support systems for medical staff (118).

**OC10-Ethical and regulatory perspectives:** In Saudi Arabia, as in many countries, concerns arise about how AI impacts patient privacy, especially regarding sensitive health data (119). AI literacy must include training on compliance with Saudi-specific regulations, like the Health Sector Cybersecurity Framework (HSCF), which focuses on safeguarding patient data (120). The Saudi Data and AI Authority (SDAIA) plays a significant role in ensuring AI’s ethical use, setting guidelines that healthcare supply chains must follow to protect patient privacy and ensure transparency in decisions (121).

**OC11-Sustainability and scalability:** While pilot AI projects in Saudi healthcare have shown success, scaling them across the entire public health system remains a challenge (90). AI literacy programs must focus on scalability, helping professionals learn how to expand AI solutions from pilots to wider use cases (122).

## 3 Methodology

The research used a qualitative analysis employing the T-O-E framework to identify the adoption challenges of AI literacy.

The Technology-Organization-Environment (T-O-E) framework was chosen for this study as it provides a holistic perspective on AI literacy adoption in public healthcare by considering multiple influencing factors. Unlike individual-centric models such as the Technology Acceptance Model (TAM) (123) and the Unified Theory of Acceptance and Use of Technology (UTAUT) (124), which primarily focus on user perceptions and behavioral intent, the T-O-E framework captures broader institutional and contextual determinants. Drazin (125) emphasize that the T-O-E model effectively integrates technological capabilities, organizational structures, and environmental influences, making it particularly useful for studying technology adoption at the institutional level. Moreover, Baker (126) highlights that the framework is adaptable across various industries, including healthcare, due to its ability to incorporate external policy and regulatory constraints. Given that AI literacy adoption in public healthcare is shaped by multi-level barriers—including technological capabilities, organizational readiness, and external policy dynamics, the T-O-E framework provides a comprehensive analytical lens for this study.

To ensure depth and relevance of data, the study strategically selected 10 experts from the healthcare sector based on strict inclusion and exclusion criteria. Experts were included if they held decision-making roles in healthcare and AI technologies, possessed at least 5 years of experience in healthcare technology integration, and worked directly in healthcare settings. Those primarily involved in unrelated IT services or administrative roles, or with less than 5 years in the field, were excluded. Authors have deployed Best-Worst Method (BWM) approach to identify the critical AI adoption challenges in public health systems. BWM is a multi-criterion decision making method that rates the options according to the ordinal ranking of criteria based on the relative importance of the criteria (127). Depending on the objectives and environments of decision makers, the most preferable and least desirable criteria are selected first, followed by the pairwise comparisons that are essential for obtaining the optimal weights which also guarantee the consistency of the judgments made. The BWM has been used widely in different fields for its reliability and validity in other related areas. In supply chain and logistics, BWM assists in the selection of a supplier by ranking necessary criteria such as cost and quality (128). In energy planning, BWM assesses potential resources by their environmental influence and effectiveness (129, 130). The healthcare sectors utilize BWM to allocate resources, and the urban planners have integrated it with the priority of smart city projects allocating supports decision-making in product development evaluation of environments, and risk evaluation because the system allows the organization to sort out factors such as customer preference, social issues or risk probability (131). Due to the accurate balance of weights assigned to criteria, BWM is an ideal tool for strategic decision-making (132). The Best Worst Method (BWM) involves a structured pairwise comparison process that typically requires fewer comparisons than the Analytic Hierarchy Process (AHP) (133). Firstly, it operates by identifying the most and least important criteria from a set, thus making it a focused comparison approach that reduces the cognitive load and potential bias compared to AHP. Secondly, by specifically comparing all criteria to these two reference points (best and worst), the method effectively minimizes anchoring bias, as it anchors the scale on identified extremes rather than arbitrary judgments. Thirdly, the results obtained from BWM are generally more consistent and reliable due to its systematic reduction of comparison complexity and clearer decision framework (134–136).

### 3.1 Calculation of the weight of challenges using BWM method

In this step, the relative importance of 11 problems were determined. The stars for each criterion were made through usage of a 1–9 point scale on pairwise comparisons. To derive the weights of criteria and sub-criteria, the BWM optimization model as suggested by Razaei (127). This strategy helps decision-makers in decision-making since the results obtained are so reliable. Annexure I discussed step involved in BWM Method. Table 9 displays the Consistency Index for Best-Worst Method models with varying criteria counts.

**Table 9:** BWM consistency index.

1	2	3	4	5	6	7	8	9
0	0.44	1.00	1.63	2.30	3.00	3.73	4.47	5.23

## 4 Results

The medical manufacturers, healthcare service providers, and academic (Professor) were the experts who participated in this study. Participants’ demographic details are given in Table 10.

**Table 10:** Demographic details of participating experts.

Total experts	Experience	Designation	Type of role in public healthcare SC	Number of experts	Level of adoption	Percentage
10	≥15 Years	Medical Manufacturers	Producing and supplying essential medical supplies, pharmaceuticals, and healthcare equipment to ensure continuous and reliable access to necessary healthcare resources.	2	High	20%
		Healthcare service providers	Delivering medical care and services directly to patients, ensuring that resources and treatments are accessible, effectively utilized, and aligned with public health objectives.	2	Moderate	10%
		Academic (Professor)	Conducting research, developing innovative solutions, and educating future professionals, thus advancing knowledge, policy insights, and best practices that provide effective healthcare management and supply chain optimization.	2	Low	20%
	10–15 Years	Medical Manufacturers	producing and supplying essential medical supplies, pharmaceuticals, and healthcare equipment to ensure continuous and reliable access to necessary healthcare resources.	1	Moderate	10%
		Healthcare Service Providers	delivering medical care and services directly to patients, ensuring that resources and treatments are accessible, effectively utilized, and aligned with public health objectives.	2	Moderate	20%
	<10 Years	Academic (Professor)	conducting research, developing innovative solutions, and educating future professionals, thereby advancing knowledge, policy insights, and best practices that support effective healthcare management and supply chain optimization.	1	Moderate	10%

Source: Authors.

Tables 9, 10 show the best-to-others and worst-to-worst results, respectively. Weights and consistency rates are derived from a non-linear mathematical model of BWM, as presented in Tables 11, 12. Expert evaluations indicate that the BWM results demonstrate ‘C_2_’ as the most critical criterion. Experts E2, E3, E4, E7, E9, and E10 selected Criterion C_2_ as the ‘best to others’ criterion (Table 9) (Table 13).

**Table 11:** Best to others (main criteria).

Experts	Best to others	C_1_	C_2_	C_3_
		ENC	ORC	TEC
E1	ENC	1	6	3
E2	ORC	2	1	2
E3	ORC	7	1	5
E4	ORC	2	1	7
E5	TEC	5	4	1
E6	ENC	1	4	2
E7	ORC	3	1	5
E8	TEC	4	6	1
E9	ORC	2	1	4
E10	ORC	3	1	3

Experts: E1–E10; Environmental challenges, ENC (C_1_); Organizational challenges ORC (C_2_); Technical Challenges (C_3_). Table 12 illustrates the ‘others to the worst’ for the primary criteria choice.

**Table 12:** Others to the worst (main criteria) (Experts E1–E10).

Experts	Others to the worst	C_1_	C_2_	C_3_
		ENC	ORC	TEC
E1	TEC	3	4	1
E2	TEC	4	1	3
E3	ENC	1	2	6
E4	TEC	5	2	1
E5	ENC	1	4	4
E6	TEC	2	3	1
E7	ENC	1	3	6
E8	TEC	2	4	1
E9	ENC	1	3	4
E10	TEC	3	2	1

Experts: E1–E10; Environmental challenges, ENC (C_1_); Organizational challenges ORC (C_2_); Technical Challenges (C_3_). The consistency ratio was verified for all values derived from Equation 7. All values were determined to be below 0.1, which indicates the absence of any contradiction in the results (Table 13).

**Table 13:** Consistency ratio and ξ^*^ for main criteria (all experts E1–E10).

Experts	ξ^*^	CR
E1	0.098	0.032
E2	0.089	0.030
E3	0.090	0.028
E4	0.094	0.031
E5	0.060	0.020
E6	0.140	0.035
E7	0.076	0.027
E8	0.090	0.021
E9	0.089	0.024
E10	0.074	0.025

Likewise, the BWM solver was employed to calculate the weights for the sub-criteria. The global weights of the primary criteria were also computed. The ranking was conducted utilizing global weights and is shown in Table 14.

**Table 14:** Global weights.

Challenges					
Main criteria	Main criteria (Weights)	Sub-criteria	Code	Sub-criteria (Weights)	Global weights
C1-Environmental (ENC)	0.27	Ethical and Regulatory Considerations	ENC1	0.19	0.051
		Sustainability and Scalability	ENC2	0.176	0.048
C2-Organizational Challenges (ORC)	0.51	Customization and Contextualization	ORC1	0.153	0.078
		Patient-Centric Considerations	ORC2	0.192	0.098
		Interdisciplinary Collaboration	ORC3	0.122	0.062
		Cost and Resource Allocation	ORC4	0.145	0.074
		Cultural Resistance and Change Management	ORC5	0.176	0.090
		Workforce Training and Knowledge Gaps	ORC6	0.111	0.057
C3-Technical Challenges (TEC)	0.22	Data Accessibility and Quality	TEC1	0.123	0.027
		Security Concerns	TEC2	0.146	0.032
		Integration with Existing Systems	TEC3	0.178	0.684

The weights for each criterion were calculated based on the average means of the values provided by the experts. The conclusive ‘Best to others’ and ‘others to the worst’ outcomes derived from the experts for primary criteria. The scores are displayed in Table 14.

The use of the Best-Worst Method (BWM) in this research outlines the main issues arising from the development of AI literacy to support sustainable public health supply chains in Saudi Arabia. The results revealed that the type of implementation challenges in the context of organizational perspective (ORC) has the highest weight of 0.51 as the main criteria. This points to the idea that implementation Challenges such as Customization and Contextualization, Patient Centered Considerations, Cultural Resistance and Change Management, Cost and resource allocation are paramount to achievement of AI literacy. These criteria reflect the concerns closely with the contextual aspects of leveraging different AI tools in the local environment, cultural adaptation, and patient-centeredness that are crucial for creating a more significant impact of AI in the sphere. The focus on organizational Challenges goes well with the context of scope and intricate environment in the Saudi healthcare sector where engagement of stakeholders and their acceptance is most important. The research results further reveal Environmental Challenges (ENC) with a threat weight of 0.27 as another implementation challenge. Ethical standards and Regularity norms, ensuring sustainability and scalability are important concerns to be considered during the transformation planning in healthcare ecosystem. With Saudi Arabia’s Vision 2030 emphasizing technological transformation, the economic burden of AI adoption necessitates careful allocation of resources and strategic investment, especially when scaling AI projects from pilot phases to broader implementation across healthcare institutions. Technical Challenges (TEC), accounting for a weight of 0.22, emphasize the importance of Data Accessibility and Quality, Security Concerns, and Integration with Existing Systems. As AI in healthcare relies on vast and often sensitive datasets, ensuring data accessibility and quality is crucial. Moreover, the integration of AI with existing legacy systems is challenging yet essential to maximize the effectiveness of AI tools without disrupting current workflows. These technical issues, if unaddressed, could hinder the scalability and efficacy of AI-driven supply chain solutions. In addition to identifying these main challenges, the study also highlights sub-criteria with high global weights, such as Patient-Centric Considerations (0.098) and Cultural Resistance and Change Management (0.090) within the organizational challenges. These findings reinforce the importance of prioritizing stakeholder buy-in and cultural adaptability in AI literacy programs. Addressing these factors is critical for fostering trust and acceptance among healthcare professionals, patients, and other stakeholders. The consistency ratio (CR) analysis confirmed the reliability of expert judgments, with all CR values below the acceptable threshold of 0.1. This indicates a high level of agreement among experts regarding the significance of these criteria, validating the robustness of the BWM model in assessing AI literacy challenges within Saudi Arabia’s healthcare sector. Overall, the research study emphasizes the multifaceted nature of building AI literacy, involving organizational, environmental, and technical dimensions. Effective strategies must address each of these areas to foster sustainable PHSC that align with Saudi Arabia’s Vision 2030 objectives and broader Sustainable Development Goals. This research contributes to strategic and practical considerations needed to support AI integration in healthcare, laying the groundwork for further studies and the development of targeted AI literacy programs.

## 5 Conclusion and future research directions

### 5.1 Conclusion

The strategic roadmap for AI implementation in PHS, based on the TOE framework, provides a comprehensive approach to fostering a resilient and sustainable healthcare ecosystem in alignment with Saudi Vision 2030 and SDGs. By addressing organizational, environmental, and technical dimensions, this framework outlines essential actions to overcome the critical barriers to AI adoption. Organizational strategies emphasize cultural adaptation, stakeholder engagement, and literacy development; economic strategies focus on resource allocation, public-private partnerships, and scalable pilot projects; and technical strategies highlight data governance, interoperability, and cybersecurity. Collectively, these elements lay the foundation for a robust, inclusive, and sustainable healthcare AI infrastructure that supports national and global health objectives.

### 5.2 Limitations

Besides the strengths of this research framework, the study has several limitations. First, the study’s focus on Saudi Arabia limits the direct applicability of findings to other contexts with different cultural, regulatory, and healthcare environments. Second, while the TOE framework provides a structured approach, the complex and evolving nature of AI technology means that some aspects, especially regulatory compliance and cybersecurity, may require continuous updates. Additionally, the study relies on existing data and stakeholder insights, which may not fully capture the dynamic challenges and emerging technologies that could influence future AI adoption in healthcare.

### 5.3 Areas for future research

Future research should explore longitudinal studies to assess the long-term impacts of AI implementation within Saudi Arabia’s healthcare system, especially concerning patient outcomes and healthcare provider efficiency. Comparative studies between different regions and countries could offer further insights into how the TOE framework might be adapted to diverse contexts. Moreover, further research could investigate specific technical challenges such as data privacy, algorithmic transparency, and the integration of AI with emerging technologies like blockchain and the Internet of Medical Things (IoMT). Additional studies on the socio-cultural impacts of AI in healthcare focusing on ethical considerations, patient perceptions, and workforce adaptation would enrich our understanding of sustainable AI adoption in Saudi Arabia and beyond.

Table 15 discussed Future research on AI literacy adoption in public healthcare can explore several key areas:

**Table 15:** Future research directions.

Future research area	Description
Longitudinal studies on AI literacy adoption	Future studies could conduct longitudinal analyses to assess how AI literacy evolves over time within healthcare institutions, considering factors such as policy changes, technological advancements, and workforce training.
Comparative cross-country analysis	Given the varying levels of AI adoption across different healthcare systems, comparative studies across countries or regions could provide insights into how contextual factors influence AI literacy adoption.
Integration with other technology adoption models	While this study uses the T-O-E framework, future research could integrate it with models like the Diffusion of Innovation (DOI) or Institutional Theory to explore additional dimensions of AI literacy adoption.
Impact of AI literacy on healthcare outcomes	Empirical studies could investigate how improved AI literacy among healthcare professionals translates into better patient care, operational efficiency, and overall healthcare system performance.
Role of ethical and regulatory frameworks	Future research can examine how ethical concerns, data privacy regulations, and legal frameworks influence AI literacy adoption and implementation in public healthcare settings.
Personalized AI literacy training programs	Research could explore AI-driven personalized training programs for healthcare professionals, assessing their effectiveness in improving AI literacy levels and practical application.

Source: Authors.

Regulatory compliance and governance are essential to ensuring adherence to data protection for safeguarding patient data while promoting ethical AI deployment. Future research should explore strategies for effective deployment in clinical settings and enhance regulatory frameworks that balance innovation with privacy concerns. Public-private collaboration is also crucial in shaping AI ethics, with studies needed to examine how cooperation between policymakers, healthcare providers, and AI developers can establish standardized governance frameworks for ethical AI deployment. Past studies including, Dwivedi et al. (137) highlight institutional barriers to AI adoption and advocates for a multi-stakeholder approach to AI literacy, while Morley et al. (104) stresses the significance of interdisciplinary AI training to mitigate ethical risks in AI-driven healthcare. Reddy et al. (138) examine AI literacy gaps among clinicians and propose structured AI education programs to bridge these deficiencies. Future research should expand upon these perspectives, exploring region-specific AI literacy initiatives that address the unique challenges of healthcare systems while ensuring alignment with global best practices. By integrating these insights, future studies can contribute to a more comprehensive understanding of AI literacy, ethics, and governance, ultimately fostering responsible AI adoption in healthcare.

### 5.4 Strategic framework

**Figure 2:**
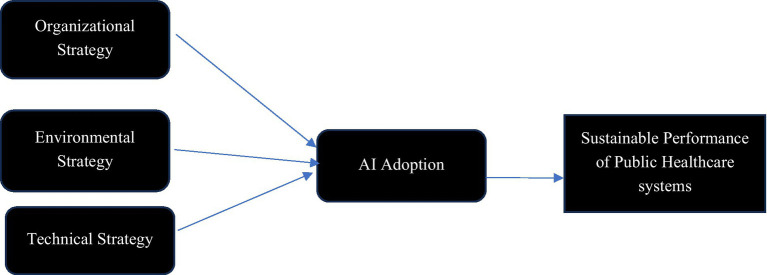
Pillars of AI adoption for sustainable performance of public healthcare systems.

For implementing AI in Saudi Arabia’s public healthcare systems, a strategic roadmap grounded in the TOE (Technology-Organization-Environment) framework is recommended. This roadmap promotes an inclusive and sustainable environment consistent with Saudi Vision 2030 and the Sustainable Development Goals (SDGs). The framework is designed to foster a resilient, inclusive, and sustainable ecosystem by addressing technological, organizational, and environmental aspects, thus overcoming key barriers identified in the study. These strategic pillars, Organizational Strategy, Environmental Strategy, and Technical Strategy, target the essential enablers and barriers to AI adoption, as illustrated in Figure 2. The strategic AI adoption leads to Sustainable performance of public healthcare systems.

The technical dimension addresses the need for robust data governance frameworks to ensure compliance with local regulations, facilitate secure data sharing, and maintain interoperability across healthcare entities. This approach supports SDG 9 by fostering innovation and infrastructure resilience. Enhanced cybersecurity and phased integration with existing systems are crucial to secure sensitive data, promoting sustainable and resilient healthcare aligned with the SDGs. From an organizational perspective, AI solutions should be adapted to Saudi Arabia’s healthcare structure and cultural context.

This involves co-designing AI tools with input from patients and healthcare providers to enhance care delivery, aligning with SDG 3 on health and wellbeing. The strategy includes comprehensive training to build AI literacy among healthcare professionals, along with stakeholder engagement through workshops and awareness initiatives to mitigate cultural resistance and ensure sustainable adoption. In terms of the economic dimension, resource allocation and partnerships with local and international technology firms, along with Public-Private Partnerships (PPPs), are vital. These partnerships provide the financial and technical support needed to scale AI adoption sustainably. By supporting pilot projects and phased expansions, these initiatives align with SDG 17, emphasizing partnerships for sustainable development. Through the TOE framework, this roadmap provides actionable strategies that uphold Saudi Arabia’s goals for a technologically advanced, sustainable healthcare ecosystem, contributing meaningfully to the nation’s broader Vision 2030 and SDG commitments.

## Data Availability

The raw data supporting the conclusions of this article will be made available by the authors without undue reservation.

## Annexure I

Step 1: The experts outline and give details of a set of parameters.

Step 2: Evaluation of super-optimal (B) and unsuitable (W) criteria by the specialists.

Step 3: A pairwise comparison has been carried out for each criterion using a 9-point scale to determine the preferred criterion in relation to others. The comparative results are illustrated as a vector ordered from the highest to the other vectors. $A_{B}=\left\{a_{B1},a_{B2},\ldots \ldots a_{Bj}\ldots a_{Bn}\right\}$

where $a_{Bj}$ represents the preferences of the best criterion *B* over the criterion *j*.

Step 4: The preferences for the remaining criteria in relation to the least favorable criterion are determined using a vector scale of 1 to 9.

$$A_{W}=\left\{a_{1W},a_{2W},\ldots \ldots a_{jW},..a_{nW}\;\right\}$$

where $a_{jW}$ denotes the preference of criteria j over the least favorable criterion W.

Step 5: Mathematical model 1 is utilized to compute the weights of the criterion (Equation 1).

(*w_1_^*^, w_2_^*^, … w_n_^*^*)

Model 1

(1)
$$\min\underset{j}{ma}x\left\{{\mathrm{||,W}}_{B}-a_{Bj}W_{j}||,||,|W_{j}-a_{jW}W_{W}|\right\}$$

s.t:

$$\frac{\sum }{j}W_{j}=1,W_{j}\geq 0\;for\;\mathrm{all}\;j$$

To determine the weigts of the criteria (*w_1_^*^, w_2_^*^, … w_n_*^*^), model 1 can be converted into model 2 (Equation 2),

Min ξ

s.t.

(2)
$$|\frac{w_{B}}{w_{j}}-a_{Bj}|\leq \xi \;for\;\mathrm{all}\;j$$

$$|\frac{w_{j}}{w_{W}}-a_{j}|\leq \xi \;for\;\mathrm{all}\;j$$

$$\frac{\sum }{j}W_{j}=1,W_{j}\geq 0\;for\;\mathrm{all}\;j$$

The consistency ratio (CR) of the Best-Worst Method (BWM) is denoted by ξ*, and the associated consistency index (CI) values are calculated using Equation 3.

(3)
$$CR=\frac{\xi^{*}}{CI}$$

A smaller ξ* corresponds to a lower CR value and greater consistency among the vectors.
